# Internet addiction is associated with differences in static and dynamic hippocampal functional connectivity in young adults: the mediating role of emotion regulation difficulties

**DOI:** 10.3389/fpsyt.2026.1903951

**Published:** 2026-07-30

**Authors:** Yue Pan, Xiang Yu, Zi-Liang Wang, Jun Zhou

**Affiliations:** 1 Department of Tourism and Humanities, Zhenjiang College, Zhenjiang, Jiangsu, China; 2 Zhenjiang Mental Health Center, Zhenjiang Fifth Affiliated Hospital of Jiangsu University, Zhenjiang, China

**Keywords:** dynamic functional connectivity, emotion regulation, hippocampus, internet addiction, static functional connectivity

## Abstract

**Background:**

Internet addiction (IA) has emerged as a pervasive public health issue among young adults, frequently accompanied by severe emotion regulation difficulties. However, the precise static and dynamic functional architecture of the hippocampus and its interplay with emotion dysregulation in IA remain incompletely understood.

**Methods:**

This study enrolled 45 young adults with IA and 29 healthy controls (HC). Utilizing resting-state functional magnetic resonance imaging (fMRI), we computed both static functional connectivity (sFC) and dynamic functional connectivity (dFC) seeded from the rostral hippocampus. Partial correlation analyses were conducted to examine the associations between aberrant connectivity and clinical symptoms. Furthermore, we investigated whether emotion dysregulation mediates the relationship between altered hippocampal connectivity and internet addiction severity.

**Results:**

Compared to HCs, individuals with IA exhibited significantly increased sFC between the left rostral hippocampus and nodes of the default mode network (DMN; precuneus and inferior parietal lobule). Furthermore, the IA group displayed heightened temporal variability between the bilateral hippocampus and visual-temporal regions (superior temporal pole, lingual gyrus, middle, and superior occipital gyri). These aberrant sFC and dFC were significantly and positively correlated with IA severity and emotion regulation difficulties. Mediation analyses further revealed that emotion dysregulation significantly and partially mediated the relationship between altered hippocampal connectivity and internet addiction severity.

**Conclusions:**

Static hyper-connectivity with the DMN and dynamic instability with visual-temporal networks constitute distinct neurobiological signatures of IA. Crucially, emotion dysregulation bridges these hippocampal network anomalies with addictive behaviors, highlighting targeted emotion regulation interventions as a promising therapeutic strategy for internet addiction.

## 1 Introduction

Internet addiction (IA) has emerged as a pervasive public health issue among young adults, characterized by compulsive internet use despite severe negative psychosocial consequences (1, 2). Growing clinical evidence indicates that individuals with IA frequently suffer from psychiatric comorbidities, particularly depression, anxiety, and profound difficulties in emotion regulation (3, 4). However, despite the escalating prevalence of IA, the precise neurobiological mechanisms driving the interplay between brain functional abnormalities and emotional dysregulation in this population remain incompletely understood (5, 57).

Behavioral addictions, including IA, are increasingly conceptualized as disorders of emotion dysregulation (6). According to contemporary theoretical frameworks, individuals often utilize compulsive internet behaviors as a maladaptive coping mechanism to alleviate negative affect or psychiatric distress (7). At the neurobiological level, this emotional regulatory failure is frequently underpinned by disruptions in large-scale brain networks responsible for affective processing and cognitive control (8). Therefore, it suggests that aberrant neural circuitry not only directly drives addictive behaviors but also deeply impairs the individual’s psychological capacity to process emotions, creating a vicious cycle of dependence.

Previous neuroimaging research has extensively explored the neural underpinnings of IA, largely focusing on the reward circuitry and cognitive control networks (9, 58). However, the hippocampus, a pivotal structural hub orchestrating learning, contextual memory, and emotional processing, has received relatively limited attention in the context of behavioral addictions (10–12). Recent study has found that reduced hippocampal volume and increased functional connectivity (FC) between hippocampal and frontal, cingulate, and temporal regions in people with ketamine use, suggesting that hippocampal abnormalities may contribute to the maintenance of addictive behaviors through altered memory and emotional processing (13). Consistent with these observations in substance use, converging evidence from behavioral addictions has also implicated hippocampal dysregulation. For instance, Zhang et al. demonstrated that individuals with Internet gaming disorder exhibited increased FC between the orbital frontal cortex and the hippocampus (14).

The rostral hippocampus is anatomically distinct, sharing robust reciprocal connections with the amygdala, prefrontal cortex, and the default mode network (DMN) (15, 16). This unique connectivity profile positions the rostral hippocampus as a crucial node for affective regulation and the conditioning of addiction-related memories (17, 18). In line with this, emerging evidence suggests that hippocampal dysfunction profoundly compromises emotional processing across addictive disorders (6, 59). Aberrant hippocampal functioning could therefore impair an individual’s ability to process negative emotions, potentially triggering compulsive internet seeking as a maladaptive coping strategy (19). Beyond emotion regulation, recent systematic evidence has identified early maladaptive schemas, particularly those related to emotional deprivation, insufficient self-control, and abandonment, as significant psychological vulnerability factors for internet addiction (20). These schemas may represent deeper cognitive-affective structures that predispose individuals to maladaptive coping behaviors, potentially through the hippocampal-DMN circuitry implicated in self-referential processing and autobiographical memory.

Although these conceptual frameworks underscore the pivotal role of such large-scale neural circuits, the vast majority of existing fMRI studies on IA have relied on static functional connectivity (sFC), which assumes that neural interactions remain constant throughout the scanning period (21). The human brain, however, is a highly dynamic system. Dynamic functional connectivity (dFC) analyses, utilizing sliding-window approaches, can capture time-varying temporal fluctuations in macroscopic neural network synchronization (22–24). For instance, aberrant dynamic coupling between the amygdala-hippocampal complex and visual networks has been identified as a critical mediator between cognitive impairment and addiction severity (60). Similarly, research on substance dependence, such as in cigarette smokers, has demonstrated that evaluating dFC variability can unveil distinct connectivity alterations, particularly within the frontoparietal control network and insula, that closely track dependence severity (25). Therefore, evaluating both sFC and dFC simultaneously could provide a more comprehensive neural fingerprint of IA, particularly in understanding how the temporal instability of neural circuits contributes to affective and behavioral symptoms (26, 27).

Crucially, while previous studies have independently established links between abnormal functional connectivity, emotion dysregulation, and IA severity, the directional or mediating relationships among these factors are rarely empirically tested (28, 29). Recent research has provided empirical support for the mediating role of emotion dysregulation (30). For example, altered functional connectivity of the amygdala has been shown to significantly correlate with both emotion dysregulation and symptom severity in problematic smartphone use, suggesting that emotion-related neural circuit alterations underpin the relationship between emotional processing deficits and problematic use (31). Based on the self-medication hypothesis of addiction, we postulated that functional anomalies in the emotion-processing brain regions might exacerbate emotion regulation difficulties, which in turn drive severe internet addiction behaviors (31; Zhao et al., 2025).

To address these gaps, the present study employed seed-based sFC and dFC analyses focusing on the rostral hippocampus in young adults with IA and healthy controls (HC). We hypothesized that: (1) individuals with IA would exhibit altered static and dynamic connectivity between the rostral hippocampus and extensive cortical regions compared to HC; (2) these altered neuroimaging metrics would significantly correlate with clinical symptoms; and (3) emotion regulation difficulties would serve as a critical mediator bridging the gap between aberrant hippocampal functional connectivity and IA severity.

## 2 Methods

### 2.1 Participants

This research enrolled 76 young adults residing in Zhenjiang, Jiangsu Province, China. Eligible participants had to be right-handed native Chinese speakers between the ages of 18 and 25. Individuals were strictly excluded if they had any history of psychiatric conditions according to DSM-5 criteria (such as anxiety, mood, or substance use disorders), suffered from severe physical illnesses, or possessed any contraindications for magnetic resonance imaging. All procedures were cleared by the Ethics Committee of the Zhenjiang Mental Health Center, and every subject gave written informed consent before taking part in the study.

### 2.2 Psychometric measures

To determine the degree of internet addiction, this study utilized the 20-item Internet Addiction Test (IAT) (32). Participants responded on a 5-point Likert scale ranging from 1 (rarely) to 5 (always). Following established literature, a threshold exceeding 50 points was applied to classify participants with internet addiction, where elevated scores represent stronger addictive tendencies (33). Furthermore, levels of depression and anxiety were measured by administering the Patient Health Questionnaire-9 (PHQ-9) (34) and the Generalized Anxiety Disorder-7 (GAD-7) (35), respectively, with higher total scores reflecting greater symptom severity. Additionally, emotion regulation difficulties were measured using the 16-item Brief Difficulties in Emotion Regulation Scale (DERS-16) (36). Items on the DERS-16 are rated on a 5-point Likert scale ranging from 1 (almost never) to 5 (almost always), with higher accumulated scores indicating greater emotion dysregulation.

### 2.3 MRI data acquisition and preprocessing

Imaging was conducted on a 3.0T Philips Ingenia scanner while participants rested with their eyes closed, maintaining wakefulness. Resting-state fMRI utilized an EPI sequence (TR/TE = 2000/30 ms, flip angle = 90°, FOV = 240 × 240 mm², matrix = 64 × 64, 33 slices, thickness/gap = 3.5/0.7 mm, 240 volumes). High-resolution T1-weighted anatomical images were acquired using TR/TE = 6.31/2.9 ms, flip angle = 8°, FOV/matrix = 256 × 256, and 1 mm isotropic slices with no gap.

Data preprocessing was performed via the DPABI toolbox (37). For T1 weighted data, preprocessing included skull-stripping (BET), co-registration to the mean functional volume, tissue segmentation into gray matter, white matter (WM), and cerebrospinal fluid (CSF), and structural spatial normalization to the MNI space via the DARTEL tool. For the functional data, the standard pipeline was applied, including removal of the initial 10 volumes, slice-timing and motion correction, nuisance covariate regression (WM and CSF signals), MNI space normalization, spatial smoothing (4-mm FWHM), and temporal band-pass filtering (0.01–0.1 Hz).

### 2.4 Quality control

All MRI data underwent rigorous quality control, including visual inspections for artifacts, structural abnormalities, and the accuracy of co-registration and spatial normalization. To mitigate motion artifacts, a strict exclusion criterion of mean framewise displacement (FD) > 0.2 mm was applied, leading to the exclusion of two participants.

### 2.5 Static and dynamic functional connectivity

The primary region of interest (ROI) was defined as the rostral hippocampus, localized utilizing the Brainnetome Atlas (38). After extracting the averaged BOLD signals from this targeted seed, we mapped its functional connections with all other voxels across the brain via Pearson correlation analyses. To guarantee a normal distribution for downstream statistical testing, the raw correlation values (r) were subsequently converted into z-scores through Fisher’s z-transformation.

To assess time-varying connectivity patterns, we adopted a sliding-window framework (39, 40). Specifically, the resting-state scan was segmented into 61 consecutive windows. Each temporal window spanned 100 seconds (50 TRs) and advanced in increments of 6 seconds (3 TRs) (40). Within every isolated segment, we computed the seed-to-whole-brain functional connections for the rostral hippocampus, mirroring the static methodology. Finally, the dFC metric was operationalized at the single-voxel level by calculating the standard deviation of these window-specific z-scores across all 61 segments (41).

### 2.6 Statistics analysis

#### 2.6.1 Demographic and clinical characteristics

Demographic and clinical data were evaluated utilizing SPSS (version 25.0) and MATLAB (R2023a). To assess differences between the IA and HC, independent two samples t-tests were applied for continuous variables, while Chi-square tests were employed to compare categorical data.

#### 2.6.2 Functional connectivity analysis

Regarding the neuroimaging findings, inter-group alterations in the seed-based static and dynamic FC maps associated with the hippocampus were examined via two-sample t-tests, with the demographic variables (age, sex, and educational attainment) alongside head motion (mean FD) as covariates. Statistical significance was established using Gaussian Random Field (GRF) theory for multiple comparison correction (voxel-wise *p* < 0.001 and cluster-level *p* < 0.01, two-tailed).

#### 2.6.3 Correlation analysis

Following the inter-group comparisons, averaged sFC and dFC signals were extracted from the surviving significant clusters. We subsequently executed partial correlation models to explore the relationships between these aberrant connectivity metrics and clinical severity scales (IAT, PHQ-9, GAD-7, and DERS-16 scores) across the entire sample. These analyses inherently controlled for sex, age, years of education, and mean FD. To rigorously account for multiple testing, all corresponding p-values were adjusted via the False Discovery Rate (FDR) procedure.

#### 2.6.4 Mediation analysis

To further elucidate the underlying neuropathological mechanisms, a mediation model was constructed to investigate whether emotion regulation difficulties mediated the relationship between altered brain connectivity and internet addiction severity. This analysis was operationalized utilizing the PROCESS (Model 4) in SPSS. Specifically, the extracted FC values from significant clusters served as the independent variable (X), DERS-16 scores as the mediating variable (M), and IAT scores as the dependent variable (Y), adjusting for age, sex, education, and mean FD as covariates. The significance of the indirect effect was estimated using a non-parametric bootstrapping approach with 5,000 resamples. A mediation effect was considered statistically significant if the 95% confidence interval (CI) did not include zero.

## 3 Results

### 3.1 Participant characteristics

The final dataset comprised 74 subjects, categorized into the IA group (n = 45; mean age = 21.16 ± 2.21 years) and the HC group (n = 29; mean age = 22.00 ± 2.09 years). Baseline comparisons revealed that the two groups were well-matched, with no statistical differences regarding age (*t* = 1.642, *p* = 0.105), gender distribution (*χ^2^* = 0.059, *p* = 0.808), educational attainment (*t* = 0.827, *p* = 0.411), or head motion profiles (*t* = -0.092, *p* = 0.927). Conversely, clinical assessments demonstrated that individuals with IA had markedly elevated scores across internet addiction (*t* = -11.445, *p* < 0.001), anxiety (*t* = -4.143, *p* < 0.001), and depressive symptoms (*t* = -3.525, *p* < 0.001) (**Table 1**).

**Table 1:** Demographic and clinical characteristics between IA and HC.

Characteristics	IA (n = 45)	HCs (n = 29)	*t/χ^2^*	*p*
Gender (Female/Male)	33/12	22/7	0.059	0.808
Age (years)	21.16 (2.21)	22.00 (2.09)	1.642	0.105
Education (years)	14.84 (1.28)	15.10 (1.37)	0.827	0.411
Head motion (FD)	0.10 (0.05)	0.10 (0.04)	-0.092	0.927
IAT	61.58 (8.48)	39.41 (7.56)	-11.445	< 0.001
DERS-16	36.29 (9.64)	27.38 (7.73)	-4.18	< 0.001
GAD-7	11.58 (3.45)	8.59 (2.23)	-4.143	< 0.001
PHQ-9	15.04 (4.26)	11.90 (2.77)	-3.525	< 0.001

Values are presented as n or mean (SD). IAT, Internet Addiction Test; DERS-16, Difficulties in Emotion Regulation Scale; PHQ-9, Patient Health Questionnaire-9; GAD-7, Generalized Anxiety Disorder-7; IA, internet addition; HC, health controls; FD, framewise displacement.

### 3.2 Significant alterations in static and dynamic functional connectivity

**Figure 1:**
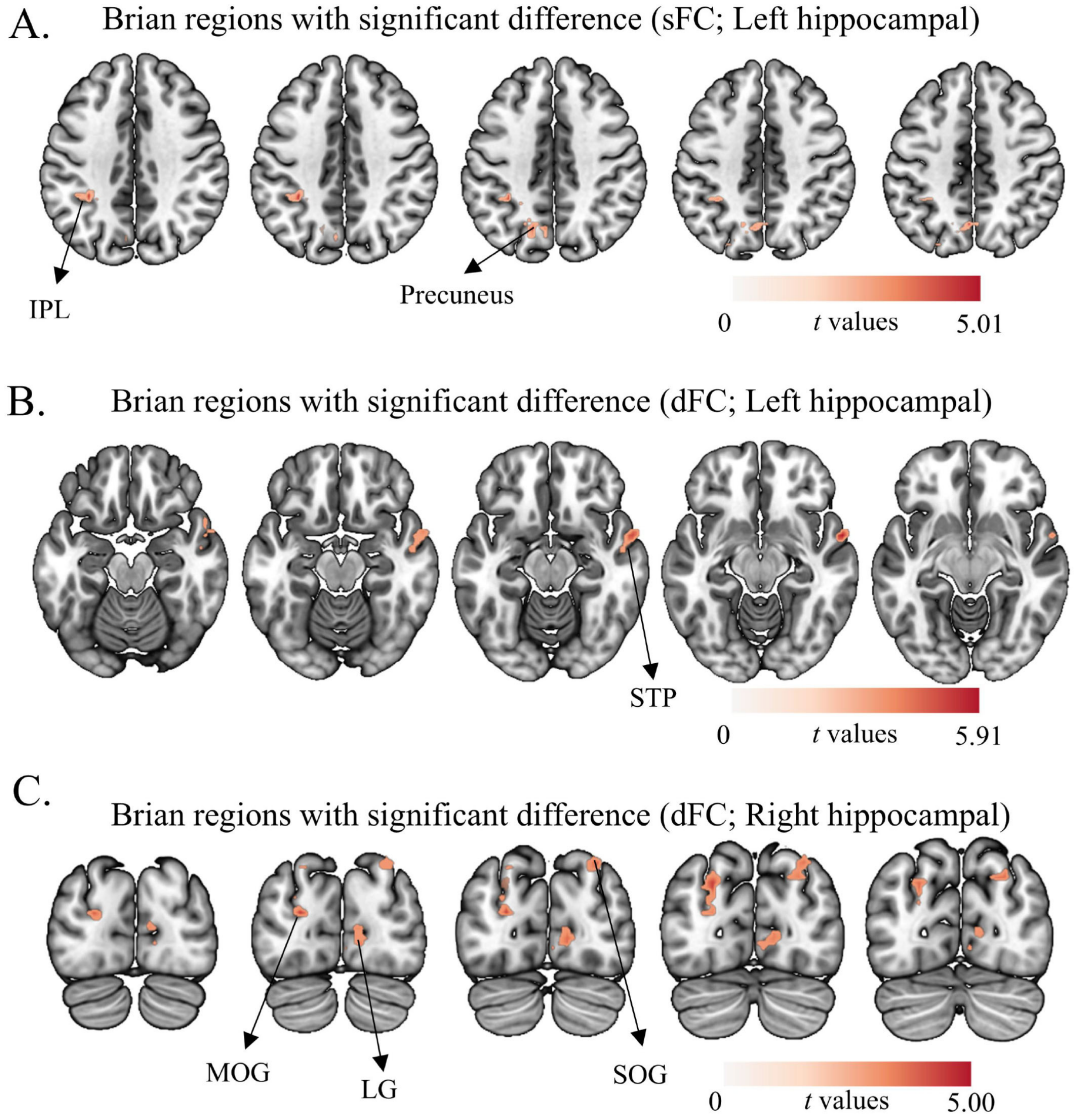
Brain regions exhibiting significant group differences in static and dynamic functional connectivity between the IA and HC groups. **(A)** Significant alterations in sFC seeded from the left hippocampus. **(B)** Significant alterations in dFC seeded from the left hippocampus. **(C)** Significant alterations in dFC seeded from the right hippocampus. The color bars represent t-values derived from voxel-wise two-sample t-tests, with warmer colors indicating increased connectivity or temporal variability in the IA group relative to HC. IPL, inferior parietal lobule; STP, superior temporal pole; MOG, middle occipital gyrus; LG, lingual gyrus; SOG, superior occipital gyrus.

Voxel-wise comparisons revealed significant inter-group differences in both static and dynamic functional connectivity seeded from the hippocampus (**Table 2**; **Figure 1**). Regarding the sFC, compared to the HC group, individuals with IA exhibited significantly increased positive connectivity between the left hippocampus and specific cortical regions, including the left precuneus (peak MNI: -3, -63, 45; peak *t* = 5.01, *p* < 0.001) and the left inferior parietal lobule (IPL; peak MNI: -33, -42, 39; peak *t* = 4.59, *p* < 0.001).

**Table 2:** Brain regions exhibiting significant differences in functional connectivity between the IA and HC groups.

Measure	ROI	AAL3	MNI coordinate of peak	*T* value of peak	*P* value of peak
sFC	Left Hip	Precuneus_L	-3, -63, 45	5.01	< 0.001
		Parietal_Inf_L	-33, -42, 39	4.59	< 0.001
dFC	Left Hip	Temporal_Pole_Sup_R	60, 3, -12	5.91	< 0.001
	Right Hip	Lingual_R	6, -69, -6	5.00	< 0.001
		Occipital_Mid_L	-24, -81, 18	4.97	< 0.001
		Occipital_Sup_R	24, -75, 39	4.09	< 0.001

IA, internet addiction; HC, healthy controls; sFC, static functional connectivity; dFC, dynamic functional connectivity; ROI, region of interest; Hip, hippocampus; AAL3, Automated Anatomical Labeling atlas version 3; MNI, Montreal Neurological Institute; L, left; R, right. Positive t-values indicate increased functional connectivity or dynamic variability in the IA group relative to the HC group.

Furthermore, dFC analysis demonstrated greater temporal variability in the IA group. Specifically, using the left hippocampus as the seed, the IA group showed enhanced dFC in the right superior temporal pole (STP; peak MNI: 60, 3, -12; peak *t* = 5.91, *p* < 0.001). When examining the right hippocampus, significant dFC increases were observed in visual processing areas, encompassing the right lingual gyrus (LG; peak MNI: 6, -69, -6; peak *t* = 5.00, *p* < 0.001), the left middle occipital gyrus (MOG; peak MNI: -24, -81, 18; peak *t* = 4.97, *p* < 0.001), and the right superior occipital gyrus (SOG; peak MNI: 24, -75, 39; peak *t* = 4.09, *p* < 0.001).

### 3.3 Correlation analysis

**Figure 2:**
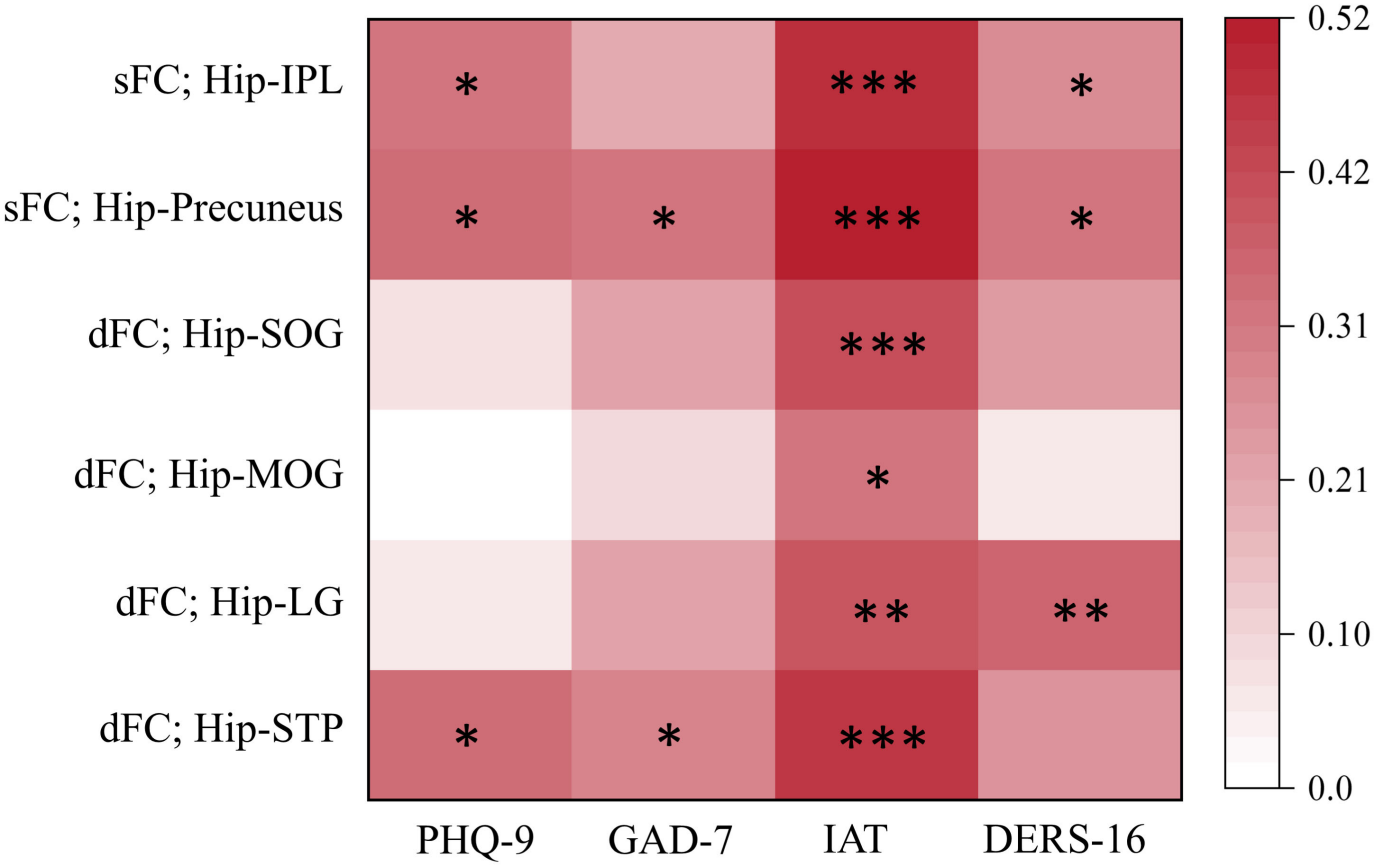
Heatmap of partial correlations between altered functional connectivity metrics and clinical severity scores. The heatmap illustrates the correlation coefficients (r) between the extracted sFC/dFC values from significant clusters and clinical measures across all participants, adjusting for age, sex, years of education, and mean framewise displacement. Asterisks denote statistical significance after FDR correction. PHQ-9, Patient Health Questionnaire-9; GAD-7, Generalized Anxiety Disorder-7; IAT, Internet Addiction Test; DERS-16, Brief Difficulties in Emotion Regulation Scale; sFC, static functional connectivity; dFC, dynamic functional connectivity. * *p_FDR_* < 0.05, ** *p_FDR_* < 0.01, *** *p_FDR_* < 0.001.

Partial correlation analyses, controlling for age, sex, years of education, and mean FD, revealed extensive significant associations between the altered functional connectivity metrics and clinical severity scores (**Figure 2**). Notably, the internet addiction severity exhibited widespread positive correlations with all identified aberrant connectivity metrics. Specifically, IAT scores were significantly associated with the sFC of the hippocampus-precuneus (*r* = 0.521, *p_FDR_* < 0.001) and hippocampus-IPL (*r* = 0.473, *p_FDR_* < 0.001). Furthermore, strong correlations were observed between IAT and the dFC of the hippocampus to the STP (*r* = 0.465, *p_FDR_* < 0.001), SOG (*r* = 0.406, *p* < 0.001), LG (*r* = 0.398, *p_FDR_* = 0.005), and MOG (*r* = 0.330, P FDR = 0.013).

Regarding emotional dysregulation and psychological distress, DERS scores showed significant positive correlations with the sFC of the hippocampus-precuneus (*r* = 0.323, *p_FDR_* = 0.014) and hippocampus-IPL (*r* = 0.271, *p_FDR_* = 0.039), as well as the dFC of the hippocampus-LG (*r* = 0.361, *p_FDR_* = 0.008). Similarly, depressive symptoms were positively linked to the sFC of the hippocampus-precuneus (*r* = 0.334, *p_FDR_* = 0.013) and hippocampus-IPL (*r* = 0.319, *p_FDR_* = 0.014), alongside the dFC of the hippocampus-STP (*r* = 0.340, *p_FDR_* = 0.013). Lastly, anxiety levels were significantly correlated with the sFC of the hippocampus-precuneus (*r* = 0.320, *p_FDR_* = 0.014) and the dFC of the hippocampus-STP (*r* = 0.282, *p_FDR_* = 0.033). All reported *p* values were adjusted using the FDR method.

### 3.4 Mediation analysis

**Figure 3:**
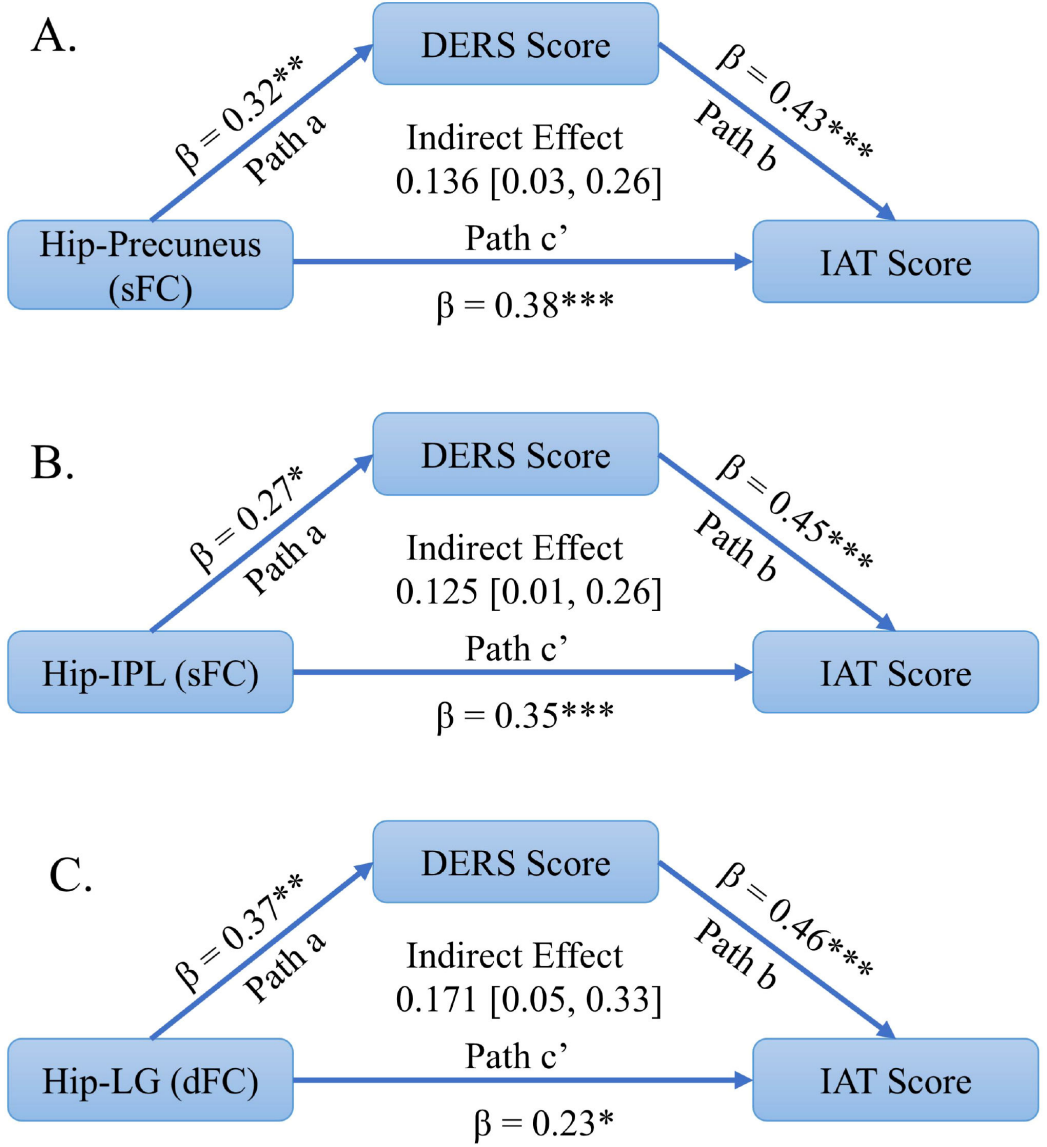
Mediation models illustrating the indirect effects of emotion regulation difficulties on the relationship between hippocampal functional connectivity and internet addiction severity. The models demonstrate that DERS scores partially mediate the associations between IAT scores and **(A)** left hippocampus-precuneus sFC, **(B)** left hippocampus-IPL sFC, and **(C)** right hippocampus-LG dFC. Standardized coefficients (β) are presented for each pathway. The completely standardized indirect effects are displayed with their 95% confidence intervals (CI). *p < 0.05, **p < 0.01, ***p < 0.001.

Based on the prerequisite correlation findings, mediation analyses were strictly conducted on the functional connectivity metrics that exhibited significant associations with both DERS and IAT scores (**Figure 3**). Consequently, three distinct mediation models were tested to determine whether emotion regulation difficulties mediated the impact of altered hippocampal connectivity on internet addiction severity.

The PROCESS macro testing (Model 4) revealed a significant indirect effect involving the static connectivity of the left hippocampus-precuneus. Specifically, the sFC metric significantly predicted DERS scores (Path a: *t* = 2.815, *p* = 0.006), which in turn significantly predicted IAT severity (Path b: *t* = 4.383, *p* < 0.001), yielding a significant indirect effect (completely standardized effect = 0.136, 95% CI: [0.032, 0.262]). The direct effect of the sFC on IAT remained significant (Path c’: *t* = 3.949, *p* < 0.001), indicating partial mediation. Similarly, significant partial mediation was established for the static connectivity of the left hippocampus-IPL. This connection significantly positively predicted DERS (Path a: *t* = 2.320, *p* = 0.023), which continuously drove IAT scores (Path b: *t* = 4.688, *p* < 0.001). The completely standardized indirect effect was 0.125 (95% CI: [0.012, 0.260]), with a significant direct effect (Path c’: *t* = 3.603, *p* = 0.001). Finally, regarding dynamic functional connectivity, the right hippocampus-LG dynamic variability significantly predicted DERS (Path a: *t* = 3.195, *p* = 0.002), which positively influenced IAT (Path b: *t* = 4.413, *p* < 0.001). This path formed a significant partial mediation model (completely standardized indirect effect = 0.171, 95% CI: [0.047, 0.325]; direct effect Path c’: *t* = 2.163, *p* = 0.034).

To evaluate whether the mediating effect of emotion dysregulation remained significant after accounting for comorbid depressive and anxiety symptoms, we repeated the mediation analyses with PHQ-9 and GAD-7 scores entered as additional covariates in the PROCESS models, while retaining age, sex, education, and mean FD as basic covariates. The indirect effects of DERS remained statistically significant for all three connectivity metrics: for the left hippocampus to precuneus sFC, the completely standardized indirect effect was 0.110 (95% CI [0.021, 0.231]); for the left hippocampus to IPL sFC, it was 0.099 (95% CI [0.010, 0.222]); and for the right hippocampus to LG dFC, it was 0.142 (95% CI [0.032, 0.289]).

## 4 Discussion

In the present study, we integrated static and dynamic resting-state fMRI approaches to investigate the functional architecture of the rostral hippocampus in young adults with IA. As hypothesized, the IA group exhibited significant functional reorganizations. Statically, we observed enhanced coupling between the left rostral hippocampus and core nodes of the DMN (precuneus and IPL). Dynamically, the IA cohort displayed increased temporal variability (dFC) between the bilateral hippocampus and regions primarily involved in visual processing and semantic integration (STP, LG, MOG, and SOG). Building upon these neuroimaging findings, our mediation analysis provided crucial evidence that emotion regulation difficulties partially mediated the specific pathways from aberrant hippocampus connectivity (specifically, sFC of the hippocampus-precuneus/IPL and dFC of the hippocampus-LG) to internet addiction severity.

Our static connectivity analysis revealed that individuals with IA had abnormally increased sFC between the left rostral hippocampus and the precuneus as well as the IPL. The precuneus and IPL are fundamental hubs of the DMN, a network heavily implicated in internally directed cognition, autobiographical memory retrieval, and self-referential processing (42, 43). These findings conceptually align with prior literature highlighting hippocampal dysregulation across diverse addictive disorders. Much like the increased hippocampal coupling with frontal and temporal regions observed in ketamine users (13), and the enhanced orbitofrontal-hippocampal connectivity seen in Internet gaming disorder (14), the hyper-connectivity within our IA sample suggests a shared neural vulnerability.

The rostral hippocampus interacts with the DMN to regulate emotional reactivity and contextualize affective experiences (44, 45). The hyper-connectivity observed in our IA sample may therefore reflect a hyperactive internally focused state, potentially underlying the rumination and psychological distress frequently reported by individuals with behavioral addictions (46, 47). This enhanced coupling might impair the brain’s ability to disengage from internet-related persistent thoughts or negative emotions, thereby continuously driving the urge to go online (48).

Beyond static alterations, we captured the temporal instability of functional networks through dFC analysis. The IA group showed increased dynamic variability between the rostral hippocampus and the STP, as well as extensive visual cortical areas including the LG, MOG, and SOG. This temporal instability echoes recent evidence showing that aberrant dynamic coupling between the amygdala-hippocampal complex and visual networks acts as a critical mediator for addiction severity(Zhao et al., 2025). Furthermore, similar to how dFC variability in the insula and frontoparietal networks closely tracks dependence severity in cigarette smokers (25), the unstable dFC profile we observed delineates a distinct signature for IA.

The STP is critically involved in socio-emotional processing and semantic memory, while the occipital visual areas are routinely activated during sensory cue-reactivity tasks in addiction research (47, 49, 50). The increased variability between the emotion-memory hub and these sensory-perceptual regions suggests an unstable, hyper-aroused neural communication pattern. This instability may render individuals with IA overly sensitive to internet-related visual cues (e.g., gaming interfaces, social media notifications), making them more susceptible to cue-induced cravings and impulsive internet use (47, 51).

Most importantly, the most compelling finding of our study is the mediating role of the DERS scores. While correlation analyses identified robust associations between brain connectivity and diverse clinical scales, the mediation models explicitly delineated a potential neuropathological pathway: altered hippocampal connectivity to emotion dysregulation to internet addiction severity. This mechanism powerfully corroborates the self-medication hypothesis of addiction postulated in our introduction (31, 52; Zhao et al., 2025). Furthermore, our results extend recent empirical work on problematic smartphone use, which identified a parallel mediating pathway involving emotion dysregulation and amygdala functional connectivity (31).

By establishing the rostral hippocampus as another crucial node in this emotion-processing circuit, we found that specifically, the abnormal static coupling within the DMN (hippocampus-precuneus/IPL) and the dynamic instability with visual regions (hippocampus-LG) both significantly predicted severe emotion regulation deficits, which subsequently exacerbated addiction severity. This mechanism strongly aligns with the psychological framework of addiction as a compensatory behavior. When young adults experience structural or functional vulnerabilities in hippocampal-cortical circuits, their capacity for top-down emotional regulation is compromised (53; Zhao et al., 2025). Consequently, they may compulsively turn to the internet as an external, highly accessible tool to modulate negative affective states, ultimately reinforcing the cycle of addiction (54, 55).

From a psychological perspective, the mediating role of emotion dysregulation indicates that IA is deeply rooted in affective vulnerabilities rather than mere behavioral excess (6, 54). Specifically, static hyper-connectivity between the hippocampus and the DMN correlates with maladaptive processing like excessive rumination (47). Conversely, dynamic instability with visual-sensory networks reflects heightened emotional reactivity to environmental cues (49). Clinically, this suggests that effective IA treatment must go beyond behavioral abstinence (56). It should explicitly target these underlying neural circuits to repair emotion regulation capacities.

Building upon this framework, our finding that emotion dysregulation mediates the relationship between hippocampal connectivity anomalies and IA severity aligns with and extends recent clinical intervention research. As Fallah et al. demonstrated, body awareness psychotherapy effectively reduces IA symptoms through improvements in both emotion regulation and executive functions (56). Notably, their mediation model revealed that emotion regulation served as a significant mediator of treatment outcomes. This convergence of evidence suggests that interventions targeting emotion regulation, whether through cognitive-behavioral approaches, mindfulness, or body awareness therapies, may exert their therapeutic effects by modulating the hippocampal-cortical circuits identified in our study. Specifically, body awareness psychotherapy might target the dynamic connectivity instabilities observed between the hippocampus and visual-sensory regions by shifting the individual’s attentional focus from external craving-related visual cues toward internal somatic sensations, thereby grounding hyper-aroused neural states and stabilizing emotion-processing networks.

### 4.1 Limitations

Several limitations should be acknowledged. First, the cross-sectional design of this study precludes causal inferences. Although our mediation models imply a directional pathway, longitudinal neuroimaging studies are essential to determine whether these hippocampal connectivity alterations are pre-existing vulnerability markers for IA or the neuroplastic consequences of prolonged internet exposure. Second, our sample consisted exclusively of young adults aged 18 to 25. Given that brain maturation, particularly in prefrontal-limbic circuits, continues throughout early adulthood, the generalizability of our findings to adolescents or older demographics remains to be verified. Third, although the present study identified significant group differences and mediation effects, the sample size (n = 45 for the IA group, n = 29 for the HC group) is relatively modest. Future studies with larger, well powered samples are warranted to replicate and extend these findings. Fourth, structural neuroimaging measures (e.g., hippocampal volume, cortical thickness) were not acquired. Such measures would have helped determine whether our functional connectivity findings reflect structural atrophy or compensatory plasticity. Future multimodal studies combining structural and functional imaging are needed to clarify structure-function coupling in IA. Fifth, the reliance on self-reported questionnaires for clinical assessments may introduce subjective reporting bias. Future studies should incorporate objective behavioral paradigms to measure emotion regulation and inhibitory control directly.

Finally, our findings open several crucial avenues for future research. In light of the observed neural alterations, future longitudinal studies should investigate whether targeted clinical interventions, such as body awareness psychotherapy, can successfully normalize the aberrant static and dynamic hippocampal connectivity patterns identified herein, potentially serving as objective neural biomarkers for treatment efficacy. Furthermore, future research should explore the “schema-connectivity hypothesis” as a potential theoretical framework. Investigating how early maladaptive schemas map onto specific static and dynamic network configurations (e.g., the hippocampal-DMN circuitry) could provide a deeper understanding of the cognitive-affective vulnerabilities driving internet addiction.

## 5 Conclusion

In conclusion, our study highlights the disrupted static and dynamic functional connectivity of the rostral hippocampus in young adults with IA. The hyper-coupling with the DMN and the dynamic instability with visual-temporal regions provide a deeper understanding of the neural substrates underlying IA. Crucially, the identification of emotion dysregulation as a significant mediator bridges the gap between brain network anomalies and addictive behaviors, suggesting that therapeutic interventions targeting emotion regulation skills may hold substantial promise for treating internet addiction.

## Data Availability

The raw data supporting the conclusions of this article will be made available by the authors, without undue reservation.
